# Genome Analysis of Cytochrome P450s and Their Expression Profiles in Insecticide Resistant Mosquitoes, *Culex quinquefasciatus*

**DOI:** 10.1371/journal.pone.0029418

**Published:** 2011-12-29

**Authors:** Ting Yang, Nannan Liu

**Affiliations:** Department of Entomology and Plant Pathology, Auburn University, Auburn, Alabama, United States of America; New Mexico State University, United States of America

## Abstract

Here we report a study of the 204 P450 genes in the whole genome sequence of larvae and adult *Culex quinquefasciatus* mosquitoes. The expression profiles of the P450 genes were compared for susceptible (S-Lab) and resistant mosquito populations, two different field populations of mosquitoes (HAmCq and MAmCq), and field parental mosquitoes (HAmCq^ G0^ and MAmCq^G0^) and their permethrin selected offspring (HAmCq^ G8^ and MAmCq^G6^). While the majority of the P450 genes were expressed at a similar level between the field parental strains and their permethrin selected offspring, an up- or down-regulation feature in the P450 gene expression was observed following permethrin selection. Compared to their parental strains and the susceptible S-Lab strain, HAmCq^G8^ and MAmCq^G6^ were found to up-regulate 11 and 6% of total P450 genes in larvae and 7 and 4% in adults, respectively, while 5 and 11% were down-regulated in larvae and 4 and 2% in adults. Although the majority of these up- and down-regulated P450 genes appeared to be developmentally controlled, a few were either up- or down-regulated in both the larvae and adult stages. Interestingly, a different gene set was found to be up- or down-regulated in the HAmCq^G8^ and MAmCq^G6^ mosquito populations in response to insecticide selection. Several genes were identified as being up- or down-regulated in either the larvae or adults for both HAmCq^G8^ and MAmCq^G6^; of these, *CYP6AA7* and *CYP4C52v1* were up-regulated and *CYP6BY3* was down-regulated across the life stages and populations of mosquitoes, suggesting a link with the permethrin selection in these mosquitoes. Taken together, the findings from this study indicate that not only are multiple P450 genes involved in insecticide resistance but up- or down-regulation of P450 genes may also be co-responsible for detoxification of insecticides, insecticide selection, and the homeostatic response of mosquitoes to changes in cellular environment.

## Introduction

Cytochrome P450s have long been of particular interest as they are critical for the detoxification and/or activation of xenobiotics such as drugs, pesticides, plant toxins, chemical carcinogens and mutagens. They are also involved in metabolizing endogenous compounds such as hormones, fatty acids, and steroids. Basal and up-regulation of P450 gene expression can significantly affect the disposition of xenobiotics or endogenous compounds in the tissues of organisms, thus altering their pharmacological/toxicological effects [1]. Insect cytochrome P450s are known to play an important role in detoxifying exogenous compounds such as insecticides [2]–[4] and plant toxins [5], [6]. While all insects probably possess some capacity to detoxify insecticides and xenobiotics, the degree to which they can metabolize and detoxify these toxic chemicals is of considerable importance to their survival in a chemically unfriendly environment [7] and to the development of resistance. A significant characteristic of insect P450s is their transcriptional up-regulation, resulting in increased P450 protein levels and P450 activities, which, in turn, cause enhanced metabolic detoxification of insecticides and plant toxins in insects, leading to the development of insecticide resistance [4], [8]–[16] and a higher tolerance to plant toxins [17], [18]. Insect P450s are also known to be an important part of the biosynthesis and degradation pathways of endogenous compounds such as pheromones, 20-hydroxyecdysone, and juvenile hormone (JH**)** [19]–[23] and thus play important roles in insect growth, development, and reproduction.

Cytochrome P450s are a superfamily that can take a number of related forms that frequently co-exist in the same cell type [24]. The rate at which a particular substrate is oxidized differs from one P450 to another, so that the overall metabolism of a specific substrate depends on the different forms present and varies between tissues, life stages, and sexes [25]. Because of the multiple cytochrome P450s expressed in each organism and the broad substrate specificity of some of these isoforms, P450s are capable of oxidizing a bewildering array of xenobiotics [25]. While the importance of P450s in insect physiology and toxicology is widely recognized, it is not yet clear how many P450 genes precisely are involved in insecticide resistance in a single insect such as the mosquito.

With the availability of the whole genome sequence for the mosquito *Culex quinquefasciatus* [26], we are now able to characterize the expression profiles of P450s in insecticide resistant mosquitoes and thus improve our understanding of the P450 gene interactions that play a role in the physiological and toxicological processes of insects. The current study focused on characterizing the expression profiles of these P450 genes from mosquito populations of *Cx. quinquefasciatu*s bearing different phenotypes in response to permethrin (susceptible, intermediate and highly resistant) in order to pinpoint the key P450 genes involved in insecticide resistance.

## Materials and Methods

### Mosquito strains

Five strains of the mosquito *Cx. quinquefasciatus* were studied. HAmCq^G0^ and MAmCq^G0^ were field resistant strains collected from Huntsville and Mobile, respectively, from sites located >600 km apart in the state of Alabama, USA in 2002; the locations were not privately-owned or protected in any way, no specific permissions were required for these locations/activities, and the study did not involve endangered or protected species. Because *Cx. quinquefasciatus* is an important urban pest in Alabama, it has been a major target for several insecticides, including Bti, malathion, resmethrin, and permethrin, and control difficulties have been reported before the collection [27]. Both Field strains had the similar levels (10-fold compared with susceptible S-Lab) of resistance to permethirn [28] and did not exposure to insecticides after established as colonies in the laboratory. HAmCq^G8^ was the 8^th^ generation of permethrin-selected HAmCq^G0^ offspring with a 2,700-fold level of resistance and MAmCq^G6^ was the 6^th^ generation of permethrin-selected MAmCq^G0^ offspring with a 570-fold level of resistance [29]. The permethrin selections for both HAmCq^G8^ and MAmCq^G6^ were performed at the 4^th^ instar larval stage [28], [29]. S-Lab was an insecticide susceptible strain provided by Dr. Laura Harrington (Cornell University).

All the mosquitoes were reared at 25±2°C under a photoperiod of 12∶12 (L:D) h and fed blood samples from horses (Large Animal Teaching Hospital, College of Veterinary Medicine, Auburn University).

### Quantitative real-time PCR (qRT-PCR)

The 4^th^ instar larvae and 2–3 day-old adults (before blood deeding) of each mosquito population had their RNA extracted for each experiment using the acidic guanidine thiocyanate-phenol-chloroform method [8]. Total RNA (0.5 µg/sample) from each mosquito sample was reverse-transcribed using SuperScript II reverse transcriptase (Stratagene) in a total volume of 20 µl. The quantity of cDNAs was measured using a spectrophotometer prior to qRT-PCR, which was performed with the SYBR Green master mix Kit and ABI 7500 Real Time PCR system (Applied Biosystems). Each qRT-PCR reaction (25 µl final volume) contained 1× SYBR Green master mix, 1 µl of cDNA, and a P450 gene specific primer pair designed according to each of the P450 gene sequences (http://cquinquefasciatus.vectorbase.org/), Table S1 with accession number for each of P450 genes) at a final concentration of 3–5 µM. All samples, including the A ‘no-template’ negative control, were performed in triplicate. The reaction cycle consisted of a melting step of 50°C for 2 min then 95°C for 10 min, followed by 40 cycles of 95°C for 15 sec and 60°C for 1 min. Specificity of the PCR reactions was assessed by a melting curve analysis for each PCR reaction using Dissociation Curves software [30]. Relative expression levels for the P450 genes were calculated by the 2^−ΔΔCT^ method using SDS RQ software [31]. The 18 S ribosome RNA gene, an endogenous control, was used to normalize the expression of target genes [15], [32], [33]. Preliminary qRT-PCR experiments with the primer pair (Table S1) for the 18 S ribosome RNA gene designed according to the sequences of the 18 S ribosome RNA gene had revealed that the 18 S ribosome RNA gene expression remained constant among all 3 mosquito strains, so the 18 S ribosome RNA gene was used for internal normalization in the qRT-PCR assays. Each experiment was repeated three to four times with different preparations of RNA samples. The statistical significance of the gene expressions was calculated using a Student's *t*-test for all 2-sample comparisons and a one-way analysis of variance (ANOVA) for multiple sample comparisons (SAS v9.1 software); a value of *P*≤0.05 was considered statistically significant. Significant overexpression was determined using a cut-off value of a ≥2-fold change in expression [34].

## Results

### Cytochrome P450 genes in *Cx. quinquefasciatus*

**Figure 1:**
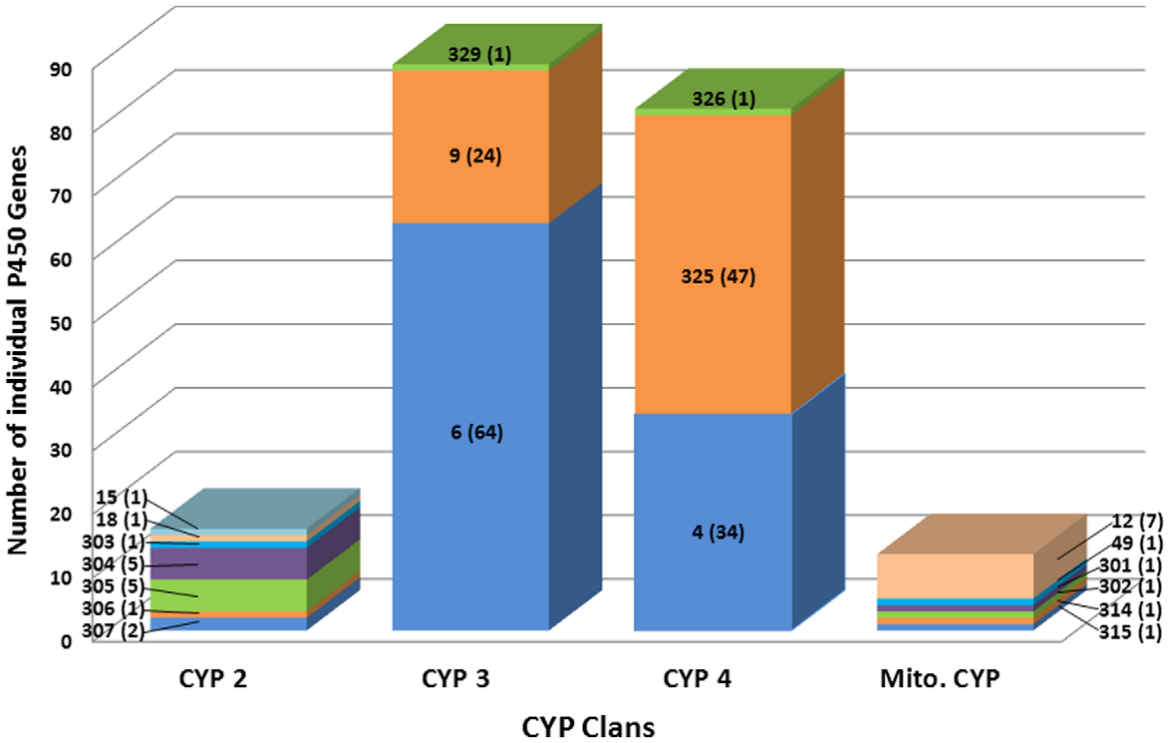
Number, family and clan distribution of cytochrome P450 genes in mosquitoes, *Culex quinquefasciatus*. The number shown along each column represents the P450 family and the number in parenthesis is the number of individual genes in the corresponding family. The P450 gene sequence information generated is from the vectorbase of the *Cx. quinquefasciatus* genome sequence (http://cquinquefasciatus.vectorbase.org/).

The *Cx. quinquefasciatus* genome sequence has revealed 204 putative P450 (CYP) genes (including 8 pseudogenes) in *Cx. quinquefasciatus* mosquitoes [26], [35], (http://cquinquefasciatus.vectorbase.org/). The *Cx. quinquefasciatus* P450s fall into four major clans of CYP2, CYP3, CYP4, and mitochondrial (Fig. 1), as do those identified in other insects [36]. Of the 204 *Cx. quinquefasciatus* P450s, the majority assemble in clans 3 and 4: 89 P450s were found in the clan CYP3, with 24 in the CYP9 family, 64 in the CYP 6 family and 1 in the CYP329 family, and 82 in the clan CYP4, with 34 in the CYP4 family, 47 in the CYP325 family, and 1 in the CYP326 family. Sixteen P450 genes were found in clan 2, with CYP families of 303 to 307, 18 and 15. The remaining 12 P450 genes were found in the mitochondrial clan with 6 P450 families of CYP12, CYP49, CYP301, CYP302, CYP314 and CYP315. Comparing this distribution with those of other insect species, Cx. quinquefasciatus showed a clear expansion of P450s in clans 3 and 4. This expanded P450 supergene family in the *Cx. quinquefasciatus* genome may provide a clue to the mechanisms that permit Culex mosquitoes to adapt to polluted larval habitats [26].

### Dynamic changes of P450 gene expression in the mosquito populations of *Culex quinquefasciatus* following permethrin selection

**Figure 2:**
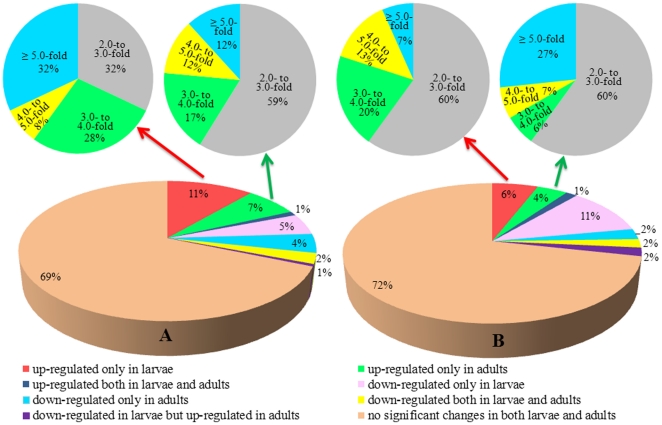
Diagrammatic representation of the analysis of the P450 gene expression profiles in both larvae and adults of permethrin selected mosquito populations HAmCq^G8^ and MAmCq^G6^ compared with their corresponding field parental populations HAmCq^G0^ and MAmCq^G0^. The statistical significance of the gene expressions was considered to be a *p* value ≤0.05. Significant overexpression was analyzed using a cut-off value of a ≥2-fold change in expression [29]. A. P450 gene expression profiles in HAmCq^G8^. B. P450 gene expression profiles in MAmCq^G6^.

To understand how the P450 gene expression profile changes following permethrin selection, we compared the gene expression of 204 P450 genes [26], (http://cquinquefasciatus.vectorbase.org/, http://drnelson.utmem.edu/CytochromeP450.html) in both larvae and adults between susceptible and resistant *Culex* mosquito populations, two different field populations of mosquitoes, and field parental mosquitoes and their permethrin selected offspring using qRT-PCR. The accession numbers of the P450 genes were listed in Table S1. Mosquito populations bearing 3 different resistance phenotypes in response to permethrin were used, ranging from susceptible (S-Lab), through intermediate resistant (HAmCq^G0^, field parental population) to highly resistant (HAmCq^G8^, 8^th^ generation permethrin selected offspring of HAmCq^G0^). Comparing the P450 gene expression profiles in both larvae and adults of permethrin selected HAmCq^G8^ mosquitoes with those of their field parental population revealed that 69% of genes were expressed at a similar level in both HAmCq^G8^ and HAmCq^G0^ (Fig. 2A), 11% were up-regulated in HAmCq^G8^ larvae compared to HAmCq^G0^, 7% were up-regulated in HAmCq^G8^ adults, 2 gene were up-regulated in both larvae and adults, 5% were down-regulated in HAmCq^G8^ larvae, 4% were down-regulated in HAmCq^G8^ adults, and 2% were down-regulated in both larvae and adults of HAmCq^G8^. Applying a cut off level of 2 [34], among the up-regulated P450 genes in larvae and adults of HAmCq^G8^, the majority were expressed at 2- to 4-fold elevated levels compared with HAmCq^G0^ and only 32% and 12% in larvae and adults, respectively, had >5-fold overexpression (Fig. 2A).

Similar expression patterns were also identified in another permethrin selected mosquito strain, Here, MAmCq^G6^, the 6^th^ generation of permethrin selected field strain of MAmCq^G0^, were compared with their parental strain of MAmCq^G0^, which was collected at a location 600 km south of the collection site for the HAmCq^G0^ mosquitoes (Fig. 2B). In MAmCq^G0^, 6% of genes were found to be up-regulated in the larvae of MAmCq^G6^ compared with those of MAmCq^G0^ and S-Lab (Fig. 2B), 4% were up-regulated in MAmCq^G6^ adults, 3 genes were up-regulated in both larvae and adults, 11% were down-regulated in larvae, 2% were down-regulated in adults, 2% were down-regulated in both larvae and adults, and 2% were down-regulated in larvae but up-regulated in adults. Taken together, these results revealed equally dynamic changes in abundance in both increased and decreased P450 gene expression in the two field mosquito strains of *Culex quinquefasciatus* following permethrin selection. Applying a cut off level of 2 [34], among the up-regulated P450 genes in larvae and adults of MAmCq^G6^ the majority exhibited 2- to 4-fold elevated levels compared with MAmCq^G0^ and only 7% and 27% in larvae and adults, respectively, of MAmCq^G6^ had >5-fold overexpression (Fig. 2B).

### P450 genes involved in up- and down-regulation in the larvae of resistant *Cx. quinquefasciatus*

Twenty five P450 genes were found to be up-regulated in the larvae stage (4^th^ larval instar) of HAmCq^G8^ mosquitoes. The expression levels of these P450 genes were ≥2-fold higher in HAmCq^G8^ than that in both S-Lab and HAmCq^G0^ mosquito strains (Table 1). The genes were distributed in clans CYP3,CYP4, and mitochondria with 7 genes in family 9, 7 in family 6, 5 in family 4, 3 in family 325, 2 in mitochondria, and 1 without anotation. Except the six P450 genes *CYP6AG12*, *CYP6AA7*, *CYP4C38*, *CYP9J35, CYP6BZ2*, and *CYP9M10* whose expression levels in parental HAmCq^G0^ mosquitoes were 2.2-, 2.8-,2.1-, 11-, 2.0- and 5-fold higher than in susceptible S-Lab mosquitoes, the expression levels of other genes were similar or lower in HAmCq^G0^ compared with the susceptible S-Lab strain (Table 1). Similar patterns were observed when comparing the changes in P450 expression in the larvae of MAmCq^G6^ with those of both the S-Lab and MAmCq^G0^ mosquito strains. Fifteen P450 genes were found to be up-regulated in the larvae of MAmCq^G6^ mosquitoes. The expression levels of these P450 genes in MAmCq^G6^ were ≥2-fold higher than those in both the S-Lab and MAmCq^G0^ mosquito strains (Table 1). These genes were distributed in clans CYP2, CYP3, and CYP4 with 7 genes in family 9, 5 in family 6, and 1 in each of families 4, 306, and 307. The expression of these genes was similar or lower in MAmCq^G0^ compared with the susceptible S-Lab strain (Table 1) except for *CYP9M10* and *CYP6AA7,* whose expression levels in the parental MAmCq^G0^ mosquitoes were 8.9- and 4.5-fold higher, respectively.

**Table 1:** Up-regulation of P450 genes in larvae of permethrin selected offspring of the field populations of *Culex quinquefasciatus.*

			Relative Gene expression ± SE^b^		
Mosquitoes	Transcript ID^a^	Gene	Parental strain^c^	Resistant strain^d^	Ratio^e^
HAmCq (25)	CPIJ002538	CYP6AG12	2.2±0.3	4.7±1.8	2.1
	CPIJ005959* #	CYP6AA7	2.8±0.9	5.9±0.6	2.1
	CPIJ003082	CYP9J42	1.3±0.2	2.9±1.5	2.2
	CPIJ001810	CYP4C38	2.1±0.6	5.1±0.3	2.4
	CPIJ015957	CYP325G4	1.2±0.2	3.3±0.3	2.8
	CPIJ005957	CYP6AA9	0.9±0.04	2.5±0.6	2.8
	CPIJ007091	CYP325Y6	1.1±0.3	3.1±1.2	2.8
	CPIJ010546*	CYP9J34	0.8±0.3	2.3±0.3	2.9
	CPIJ000926¶	-	0.7±0.1	2.2±0.2	3.1
	CPIJ016847	CYP6CQ1	0.9±0.2	2.8±0.9	3.1
	CPIJ009478	CYP4D42v1	1.3±0.1	4.1±0.9	3.2
	CPIJ010540	CYP9J35	11±2.0	39±10	3.5
	CPIJ005956	CYP6BZ2	2.0±0.6	7.3±1.1	3.7
	CPIJ010537*	CYP9J45	0.6±0.3	2.3±1.3	3.8
	CPIJ012470*	CYP9AL1	0.6±0.2	2.3±0.4	3.8
	CPIJ014218*	CYP9M10	5.0±1.2	21±4.0	4.2
	CPIJ005954	CYP6CC2	0.6±0.004	2.5±0.6	4.2
	CPIJ010225	CPY12F7	0.5±0.06	2.6±0.5	5.2
	CPIJ010227	CYP12F13	0.5±0.06	2.6±0.5	5.2
	CPIJ010543	CYP9J40	0.6±0.2	3.6±1.3	6.0
	CPIJ018943* #	CYP4C52v1	0.5±0.04	3.1±1.8	6.2
	CPIJ005955*	CYP6P14	0.6±0.1	3.8±0.6	6.3
	CPIJ001759	CYP4H40	0.3±0.09	2.1±0.6	7.0
	CPIJ020229	CYP4D42v2	0.4±0.1	2.8±2.4	7.0
	CPIJ017021	CYP325K3v1	0.2±0.02	2.1±0.2	11
MAmCq (15)	CPIJ014218*	CYP9M10	8.9±1.6	14±3.9	1.6
	CPIJ010548#	CYP9J39	0.9±0.4	2.0±0.3	2.2
	CPIJ005958	CYP6AA8	0.7±0.001	1.8±0.8	2.6
	CPIJ001039	CYP306A1	1.0±0.1	2.6±0.06	2.6
	CPIJ005959* #	CYP6AA7	4.5±1.0	12±4.2	2.7
	CPIJ005332	CYP9J43	0.9±0.08	2.5±0.02	2.8
	CPIJ004411	CYP6Z12	1.4±0.2	4.0±1.9	2.9
	CPIJ005955*	CYP6P14	1.6±0.1	4.6±0.5	2.9
	CPIJ008566	CYP6Z15	0.7±0.06	2.1±0.7	3.0
	CPIJ010546*	CYP9J34	1.1±0.4	3.4±1.1	3.1
	CPIJ010537*	CYP9J45	1.0±0.3	3.1±1.3	3.1
	CPIJ012470*	CYP9AL1	0.9±0.2	3.4±0.2	3.8
	CPIJ010544	CYP9J33	0.6±0.1	2.9±1.0	4.8
	CPIJ000989	CYP307B1	0.5±0.2	2.5±1.2	5.0
	CPIJ018943* #	CYP4C52v1	0.2±0.02	2.7±1.7	14

a The transcript ID number from the vectorbase of the *Cx. quinquefasciatus* genome sequence (http://cquinquefasciatus.vectorbase.org/).

b The relative level of gene expression represents the ratio of the gene expression in each permethrin selected strain compared with that in the susceptible S-Lab strain. The relative level of gene expression for S-Lab is 1.

c Parental strain for HAmCq population is HAmCq^G0^ with a 10-fold level of resistance to permethrin compared with S-Lab and for MAmCq is MAmCq^G0^ with a 10-fold level of resistance to permethrin [28].

d Permethrin selected strain for HAmCq population is HAmCq^G8^ with a 2700-fold level of resistance to permethrin and for MAmCq is MAmCq^G6^ with a 570-fold level of resistance to permethrin [28].

e The ratio of the relative gene expression in each permethrin selected strain compared its parental strain.

*The genes that are up regulated in both larvae of HAmCq^G8^ and MAmCq^G6^.

# The genes that are up regulated in both larvae and adults of HAmCq^G8^ and/or MAmCq^G6^.

¶ No annotation in Dr. Nelson's P450 homepage http://drnelson.utmem.edu/CytochromeP450.html.

Beside the up-regulation of P450 genes identified in the larvae of *Cx. quinquefasciatus* following permethrin selection, a number of P450 genes were found to be down-regulated in larvae of permethrin selected *Cx. quinquefasciatus*. Sixteen P450 genes were down-regulated in the larvae (4^th^ larval instar) of HAmCq^G8^ mosquitoes. The expression levels of these P450 genes in HAmCq^G8^ were ≤2-fold lower than that in HAmCq^G0^ mosquitoes (Table 2). These down-regulated genes were distributed in clans CYP3 and CYP4, with 2 genes in family 9, 10 in family 6, and 2 in each of families 4 and 325. The expression of the majority of these genes in HAmCq^G8^ was at similar or lower levels compared with that in susceptible S-Lab mosquitoes, even though most were expressed at higher levels in HAmCq^G0^ than in S-Lab (Table 2). Although the similar P450 down-regulation patterns were also found in the larvae of MAmCq^G6^ compared with both S-Lab and MAmCq^G0^, we did notice extended numbers and distribution of these genes in the CYP clans compared with HAmCq mosquitoes. Thirty P450 genes were down-regulated in the larvae (4^th^ larval instar) of MAmCq^G6^ mosquitoes. The expression levels of these P450 genes in MAmCq^G6^ were ≤2-fold lower than in that in MAmCq^G0^ mosquitoes (Table 2). The genes were distributed in clans CYP2, CYP3,CYP4, and mitochondria with 3 gene in family 9, 2 in family 6, 11 in family 4, and 8 in family 325, 1 in family 326, 2 in family 12, 1 in each of families 304 and 18, and 1 without annotation. The expression levels of these genes were again similar or lower in MAmCq^G6^ compared with susceptible S-Lab mosquitoes, even though most were expressed at higher levels in MAmCq^G0^ than in S-Lab (Table 2).

**Table 2:** Down-regulation of P450 genes in larvae of permethrin selected offspring of the field populations of *Culex quinquefasciatus.*

			Relative Gene expression ± SE^b^		
Mosquitoes	Transcript ID^a^	Gene	Parental strain^c^	Resistant strain^d^	Ratio^e^
HAmCq (16)	CPIJ009085	CYP6AG13	1.3±0.07	0.6±0.04	−2.2
	CPIJ019586§	CYP6Z13P	2.8±2.2	1.3±0.3	−2.2
	CPIJ006950	CYP325BG1	6.7±1.9	3.0±0.7	−2.2
	CPIJ016852	CYP6N19	2.9±0.9	1.0±0.3	−2.9
	CPIJ005683#	CYP325Y10	5.3±2.7	1.8±0.5	−2.9
	CPIJ008972^#^ §	CYP6F5P	2.9±0.7	0.9±0.1	−3.2
	CPIJ018716*	CYP4C38	1.0±0.06	0.3±0.1	−3.3
	CPIJ014219§	CYP9M10-de1b	2.6±0.5	0.8±0.2	−3.3
	CPIJ009473#	CYP4D41	2.1±0.8	0.6±0.01	−3.5
	CPIJ017462	CYP6E1	0.7±0.02	0.2±0.0	−3.5
	CPIJ011129	CYP6N25	3.5±1.5	0.9±0.1	−3.9
	CPIJ000299	CYP6AH3	4.8±0.7	1.1±0.2	−4.4
	CPIJ010547#	CYP9J47	1.6±0.3	0.3±0.08	−5.3
	CPIJ003377*	CYP6BY5	2.9±0.008	0.5±0.08	−5.8
	CPIJ003361	CYP6BY2	1.9±0.3	0.2±0.005	−9.5
	CPIJ003375* #	CYP6BY3	1.7±0.2	0.05±0.02	−34
MAmCq (30)	CPIJ017351	CYP4C50v1	1.4±0.3	0.7±0.07	−2.0
	CPIJ018854	CYP4C50v2	1.4±0.3	0.7±0.07	−2.0
	CPIJ010542	CYP9J38	1.2±0.2	0.6±0.2	−2.0
	CPIJ017198	CYP325BF1-de1b	1.4±0.2	0.7±0.06	−2.0
	CPIJ010228	CYP12F12	1.2±0.2	0.6±0.3	−2.0
	CPIJ017243	CYP304B4	2.3±0.4	1.1±0.2	−2.1
	CPIJ007090	CYP325Y5	2.2±0.07	1.0±0.07	−2.2
	CPIJ014579	CYP4AR3	2.1±1.2	0.9±0.08	−2.3
	CPIJ010231	CYP12F9	1.4±0.2	0.6±0.3	−2.3
	CPIJ019765#	CYP9M14	0.7±0.8	0.3±0.1	−2.3
	CPIJ007091	CYP325Y6	1.5±0.1	0.6±0.2	−2.5
	CPIJ015953	CYP325BF1v2	1.5±0.2	0.6±0.004	−2.5
	CPIJ015318	CYP325V5v2	3.3±0.5	1.3±0.09	−2.5
	CPIJ018944	CYP4C51v1	8.9±0.6	3.4±0.2	−2.6
	CPIJ003377*	CYP6BY5	2.7±0.6	1.0±0.3	−2.7
	CPIJ011843	CYP325BH1	9.8±2.5	3.6±2.5	−2.7
	CPIJ001038	CYP18A1	5.2±1.6	1.9±1.4	−2.7
	CPIJ009569	CYP326BK1	3.1±1.4	1.1±0.2	−2.8
	CPIJ001757#	CYP4H39	3.6±1.2	1.2±0.03	−3.0
	CPIJ001754	CYP4J6	4.5±0.4	1.5±0.6	−3.0
	CPIJ001755	CYP4J19	0.9±0.1	0.3±0.0	−3.0
	CPIJ009477	CYP4D19	4.3±0.7	1.4±0.6	−3.1
	CPIJ018716*	CYP4C38	1.9±0.8	0.6±0.05	−3.2
	CPIJ009475	CYP4D43	1.7±0.4	0.5±0.3	−3.4
	CPIJ014220	CYP9M12	1.4±0.1	0.4±0.1	−3.5
	CPIJ015961	CYP325BE1	2.8±0.6	0.7±0.09	−4.0
	CPIJ009471¶	-	1.2±0.07	0.2±0.04	−6.0
	CPIJ003375* #	CYP6BY3	2.8±0.06	0.3±0.01	−9.3
	CPIJ001810#	CYP4C38	16±2.5	1.4±0.3	−11
	CPIJ017200	CYP325N3v2	6.0±1.5	0.5±0.1	−12

a The transcript ID number from the vectorbase of the *Cx. quinquefasciatus* genome sequence (http://cquinquefasciatus.vectorbase.org/).

b The relative level of gene expression represents the ratio of the gene expression in each resistant strain compared with that in the susceptible S-Lab strain. The relative level of gene expression for S-Lab is 1.

c Parental strain for HAmCq population is HAmCq^G0^ with a 10-fold level of resistance to permethrin compared with S-Lab and for MAmCq is MAmCq^G0^ with a 10-fold level of resistance to permethrin [28].

d Permethrin selected strain for HAmCq population is HAmCq^G8^ with a 2700-fold level of resistance to permethrin and for MAmCq is MAmCq^G6^ with a 570-fold level of resistance to permethrin [28].

e The ratio of the relative gene expression in each permethrin selected strain compared its parental strain.

* The genes that are down-regulated in both larvae of HAmCq^G8^ and MAmCq^G6^.

# The genes that are down-regulated in both larvae and adults of HAmCq^G8^ and/or MAmCq^G6^.

¶ No annotation in Dr. Nelson's P450 homepage http://drnelson.utmem.edu/CytochromeP450.html.

§ pseudogene.

### P450 genes involved in up- and down-regulation in resistant *Cx. quinquefasciatus* adults

The expression of 204 *Culex* P450 genes in the adults of the 5 mosquito populations was examined using qRT-PCR. Seventeen P450 genes were found to be up-regulated in the adult stage (2–3 day old) of HAmCq^G8^ mosquitoes. The expression levels of these P450 genes in HAmCq^G8^ were ≥2-fold higher than that in both S-Lab and HAmCq^G0^ mosquito strains (Table 3). The overexpression levels of the up-regulated P450 genes in all the mosquito populations tested were closely correlated with their levels of resistance and were higher in permethrin-selected mosquitoes than in their parent field strain. These genes were mainly distributed in clans CYP3 and CYP4, with 3 genes in family 9, 5 in family 6, 5 in family 4, and 3 in family 325. One gene was in mitochondria clan, family 12. The expression of all these genes in HAmCq^G0^ was similar or lower than in susceptible S-Lab mosquitoes (Table 3). Similar changes in the P450 gene expression were also found in MAmCq^G6^ adults compared with their S-Lab and MAmCq^G0^ counterparts. Fifteen P450 genes were up-regulated in adult MAmCq^G6^ mosquitoes. The expression levels of these P450 genes were ≥2-fold higher than those in both S-Lab and MAmCq^G0^ adults (Table 3). As in the HAmCq^G8^ mosquitoes, the genes whose expression changed in MAmCq^G6^ mosquitoes following permethrin selection were also distributed in clans CYP3 and CYP4, with 1 gene in family 9, 2 in family 6, 1 in family 4, and 11 in family 325. The expression of these genes was similar or lower in MAmCq^G0^ compared with susceptible S-Lab mosquitoes except for *CYP325BF1v2* and *CYP325K3v1*, which were 2.4- and 3-fold higher, respectively, in MAmCq^G0^ (Table 4).

**Table 3:** Up-regulation of P450 genes in adults of permethrin selected offspring of the field populations of *Culex quinquefasciatus.*

			Relative Gene expression ± SE^b^		
Mosquitoes	Transcript ID^a^	Gene	Parental strain^c^	Resistant strain^d^	Ratio^e^
HAmCq (17)	CPIJ017199	CYP325BF1v1	0.9±0.1	1.8±0.3	2.0
	CPIJ019587	CYP6Z14	1.1±0.3	2.3±0.7	2.1
	CPIJ010536	CYP9J44	0.9±0.06	1.9±0.2	2.1
	CPIJ005959* #	CYP6AA7	1.4±0.2	3.2±1.0	2.3
	CPIJ015959	CYP325BJ1	1.9±0.1	4.3±0.2	2.3
	CPIJ015318	CYP325V5v2	0.9±0.02	2.2±0.5	2.4
	CPIJ010548*	CYP9J39	1.0±0.1	2.5±0.1	2.5
	CPIJ016284	CYP4J4	0.8±0.05	2.0±0.2	2.5
	CPIJ011127	CYP4H34	0.9±0.08	2.4±0.3	2.7
	CPIJ010203	CYP9AM1	0.9±0.1	2.7±0.6	3.0
	CPIJ001758	CYP4H38	0.7±0.4	2.2±0.7	3.1
	CPIJ009085	CYP6AG13	0.6±0.2	1.9±0.2	3.2
	CPIJ012640	CYP6CP1	0.9±0.08	3.3±1.1	3.7
	CPIJ018943* #	CYP4C52v1	0.8±0.02	3.6±2.0	4.5
	CPIJ006721	CYP4H37v1	0.6±0.05	2.9±0.8	4.8
	CPIJ003389	CYP6BY7	0.5±0.2	2.7±0.08	5.4
	CPIJ010230	CYP12F10	1.8±0.01	10±2.0	5.6
MAmCq (15)	CPIJ005957	CYP6AA9	0.9±0.09	1.9±0.4	2.1
	CPIJ015953	CYP325BF1v2	2.4±0.8	5.3±1.8	2.2
	CPIJ007092	CYP325Y7	0.9±0.01	2.1±0.4	2.3
	CPIJ015961	CYP325BE1	1.1±0.2	2.5±1.3	2.3
	CPIJ010548* #	CYP9J39	0.9±0.04	2.2±0.2	2.4
	CPIJ007091	CYP325Y6	1.3±0.3	3.2±1.7	2.5
	CPIJ007090	CYP325Y5	0.8±0.03	2.0±1.1	2.5
	CPIJ005959* #	CYP6AA7	1.3±0.5	3.4±1.0	2.6
	CPIJ015954	CYP325N3v1	0.7±0.2	1.9±0.5	2.7
	CPIJ006952	CYP325BG3	1.7±0.07	5.9±0.8	3.5
	CPIJ010272	CYP325BK2	0.9±0.07	4.1±1.0	4.6
	CPIJ014730	CYP325AA2	0.3±0.2	1.9±0.6	6.3
	CPIJ017021	CYP325K3v1	3.0±0.09	20±1.6	6.7
	CPIJ005685	CYP325BB2	0.9±0.1	6.1±2.0	6.8
	CPIJ018943* #	CYP4C52v1	0.2±0.02	2.8±0.7	14

a The transcript ID number from the vectorbase of the *Cx. quinquefasciatus* genome sequence (http://cquinquefasciatus.vectorbase.org/).

b The relative level of gene expression represents the ratio of the gene expression in each resistant strain compared with that in the susceptible S-Lab strain. The relative level of gene expression for S-Lab is 1.

c Parental strain for HAmCq population is HAmCq^G0^ and for MAmCq population is MAmCq^G0^.

d Permethrin selected strain for HAmCq population is HAmCq^G8^ and for MAmCq population is MAmCq^G6^.

e The ratio of the relative gene expression in each permethrin selected strain compared its parental strain.

*The genes that are up regulated in both adult of HAmCq^G8^ and MAmCq^G6^

# The genes that are up regulated in both larvae and adults of each of HAmCq^G8^ and MAmCq^G6^, or both.

**Table 4:** Down-regulation of P450 genes in adults of permethrin selected offspring of the field populations of *Culex quinquefasciatus.*

			Relative Gene expression ± SE^b^			
Mosquitoes	Transcript ID^a^	Gene	Parental strain^c^	Resistant strain^d^		Ratio^e^
HAmCq (14)	CPIJ008972^#^ §	CYP6F5P	0.8±0.02		0.4±0.04	−2.0
	CPIJ010547#	CYP9J47	2.0±0.01		0.9±0.2	−2.2
	CPIJ016849	CYP6M12	5.1±0.9		2.2±0.5	−2.3
	CPIJ003376	CYP6BY4	1.9±0.4		0.8±0.1	−2.4
	CPIJ005332	CYP9J43	3.2±0.1		1.2±0.2	−2.7
	CPIJ000294*	CYP4J13	1.6±0.09		0.6±0.1	−2.7
	CPIJ010542	CYP9J38	0.8±0.1		0.3±0.2	−2.7
	CPIJ006951	CYP325BG2P	1.1±0.4		0.4±0.1	−2.8
	CPIJ014730	CYP325AA2	0.6±0.2		0.2±0.1	−3.0
	CPIJ009473#	CYP4D41	3.3±0.2		0.9±0.04	−3.7
	CPIJ007093	CYP325Y8	2.3±0.3		0.6±0.06	−3.8
	CPIJ005683#	CYP325Y10	2.7±0.2		0.5±0.2	−5.4
	CPIJ010480	CYP4J20	1.1±0.2		0.1±0.04	−11
	CPIJ003375* #	CYP6BY3	0.4±0.04		0.02±0.007	−20
MAmCq (9)	CPIJ001757#	CYP4H39	1.7±0.1		0.8±0.1	−2.1
	CPIJ019765#	CYP9M14	2.3±0.4		1.1±0.3	−2.1
	CPIJ017245	CYP304B6	1.3±0.3		0.6±0.06	−2.2
	CPIJ010545	CYP9J41	1.4±0.3		0.6±0.02	−2.3
	CPIJ003375* #	CYP6BY3	0.7±0.02		0.3±0.03	−2.3
	CPIJ000294*	CYP4J13	1.3±0.2		0.5±0.05	−2.6
	CPIJ017242	CYP304C1	2.9±0.3		1.1±0.1	−2.6
	CPIJ010538	CYP9J46	0.02±0.01		0.005±0.003	−4.0
	CPIJ001810#	CYP4C38	2.3±0.1		0.1±0.02	−23

a The transcript ID number from the vectorbase of the *Cx. quinquefasciatus* genome sequence (http://cquinquefasciatus.vectorbase.org/).

b The relative level of gene expression represents the ratio of the gene expression in each resistant strain compared with that in the susceptible S-Lab strain. The relative level of gene expression for S-Lab is 1.

c Parental strain for HAmCq population is HAmCq^G0^ and for MAmCq population is MAmCq^G0^.

d Permethrin selected strain for HAmCq population is HAmCq^G8^ and for MAmCq population is MAmCq^G6^.

e The ratio of the relative gene expression in each permethrin selected strain compared its parental strain.

* The genes that are down-regulated in both adult of HAmCq^G8^ and MAmCq^G6^.

# The genes that are down-regulated in both larvae and adults of each of HAmCq^G8^ and MAmCq^G6^, or both

§ pseudogene.

As in the mosquito larvae, a number of P450 genes were down-regulated in adult *Cx. quinquefasciatus* following permethrin selection. Fourteen P450 genes were down-regulated in adult HAmCq^G8^ mosquitoes. The expression levels of these P450 genes in HAmCq^G8^ were ≤2-fold lower than that in HAmCq^G0^ strain (Table 4). These genes were distributed in clans CYP3 and CYP4, with 3 genes in family 9, 4 in family 6, 3 in family 4, and 4 in family 325. Apart from *CYP6M12*, whose expression was ∼2-fold higher in HAmCq^G8^ than in the susceptible S-Lab strain, all were expressed at lower levels in HAmCq^G8^ than in S-Lab adults even though most of the P450 genes in HAmCq^G0^ were expressed at higher levels than in S-Lab mosquitoes (Table 4). Similar down-regulation patterns for P450 were again found in MAmCq^G6^ adults compared with both S-Lab and MAmCq^G0^ adults. Nine P450 genes were down-regulated in MAmCq^G6^ mosquitoes, the expression levels of these 9 P450 genes were ≤2-fold lower in MAmCq^G6^ than that in MAmCq^G0^ mosquitoes (Table 4). The genes were distributed in clans CYP2, CYP3, and CYP4, with 2 genes in family 304, 3 in family 9, 1 in family 6, and 3 in family 4. All these genes had lower expression levels in MAmCq^G6^ than in S-Lab adults; the expression of these genes in the MAmCq^G0^ mosquitoes was similar to that in the S-Lab strain except for *CYP9J46*, whose expression was much lower (Table 4).

## Discussion

Two hundred and four putative P450 (CYP) genes in the genome of *Cx. quinquefasciatus* mosquitoes [26], [35], (http://cquinquefasciatus.vectorbase.org/) have put them in the largest P450 repertoire for any insect genome that has been reported so far; it is larger than that of *Anopheles gambiae* (111 P450s [37]), *Aedes aegypti* (160 P450s [34]), *Drosophila melanogaster* (90 P450s [38]), *Nasonia vitripennis* (jewel wasp, 92 P450s, [39]), *Bombyx mori* (silk moth, 86 P450s [40]), honeybee *Apis mellifera* (46 P450s [41]), *Tribolium castaneum* (red flour beetle, 134 P450s [42], [43]) were reported by Dr. nelson, http://drnelson.utmem.edu/CytochromeP450.html), pea aphid *Acyrthosiphon pisum* (83putative/58 complete P450, [43]), green peach aphid *Myzus persicae* (115 P450s, [43]), *Pediculus humanus* (human body louse, 37 P450s, [44]) and ants (http://drnelson.utmem.edu/CytochromeP450.html).

Our previous studies have indicated that P450s may be one of the primary enzymes involved in detoxifying permethrin and conferring permethrin resistance in *Culex* mosquitoes [45]. In order to examine the possible role of P450 genes, as a whole, in the development of insecticide resistance in *Culex quinquefasciatus* mosquitoes, we, for the first time, examined the expression profiles of a total of 204 P450 genes in both larvae and adults of *Cx. quinquefasciatus* by comparing the profiles for susceptible and resistant mosquito populations, two different field populations of mosquitoes, and field parental mosquitoes and their permethrin selected offspring. Insecticide resistance is generally assumed to be a pre-adaptive phenomenon, where prior to insecticide exposure rare individuals carrying an altered (varied) genome already exist, thus allowing the survival of those carrying the genetic variance after insecticide selection [46]. We therefore expected that the number of individuals carrying the resistance P450 genes or alleles should increase in a population following selection and become predominant under severe selection pressure. The approach adopted for this study, which compared P450 gene expression among different mosquito populations and between two parental field populations, HAmCq^G0^ and MAmCq^G0^, and their permethrin selected offspring, HAmCq^G8^and MAmCq^G6^, for different levels of insecticide resistance highlighted the importance of P450 genes in resistance by detecting the changes in their expression within each population following permethrin selection. Our results showed a dynamic change in the P450 genes expressed in both of the field mosquito strains of *Cx. quinquefasciatus* following permethrin selection. Interestingly, most of these up- and down-regulated P450 genes in *Cx. quinquefasciatus* were found to be developmentally regulated following selection: changes in the level of expression (either increasing [up-regulation] or decreasing [down-regulation]) in the larval stage of mosquitoes following the selection were not found in the adult stage and vice versa. However, several genes were identified that had up- or down-regulation patterns that not only reflected the permethrin selection but were also consistent in both the larval and adult stages of the mosquitoes, suggesting the importance of these genes in response to insecticide resistance over the mosquitoes' whole life span. Comparison of the P450 gene expression between two different field mosquito populations following permethrin selection revealed that although both mosquito populations had a similar number of the P450 genes that were up- and down-regulated, the two populations for the most part regulated a different gene set in response to the insecticide selection. However, several genes were identified as being up- or down-regulated in either the larvae or adults for both HAmCq^G8^ and MAmCq^G6^; of these, *CYP6AA7* and *CYP6BY3* were up- and down-regulated, respectively, across all the life stages and populations of mosquitoes, suggesting that these genes are indeed related to insecticide selection. These results further propose that different mechanisms and/or P450 genes may be involved in the response to insecticide pressure for different developmental stages of mosquitoes and in different populations of mosquitoes [28]; some are specific to certain development stages and others provide protection throughout the insect's life cycle.

Basal and up-regulation of P450 gene expression can significantly affect the disposition of xenobiotics or endogenous compounds in the tissues of organisms and thus alter their pharmacological/toxicological effects [1]. In many cases, increased P450-mediated detoxification has been found to be associated with enhanced metabolic detoxification of insecticides, as evidenced by the increased levels of P450 proteins and P450 activity that result from constitutively transcriptional overexpression of P450 genes in insecticide resistant insects [4], [9], [10], [13]–[16], [47]–[50]. In addition, multiple P450 genes have been identified as being up-regulated in several individual resistant organisms, including house flies and mosquitoes [12]-[14], [16], [49], thus increasing the overall expression levels of P450 genes. These findings suggest that overexpression of multiple P450 genes is likely to be a key factor governing increased levels of detoxification of insecticides and insecticide resistance. Nevertheless, although their importance in insect physiology and toxicology is widely recognized, there are gaps in our knowledge of insect P450s. One crucial piece of information that has been missing up until now is the issue of how many P450 genes are involved in insecticide resistance in a single organism, in this case the mosquito. The availability of the whole genome sequence of mosquitoes *Culex quinquefasciatus* [26] has enabled us to address this question by characterizing the expression profiles of P450s in insecticide resistant mosquitoes at a genome-wide level.

Our comparison of P450 gene expression profiles between two field mosquito populations following permethrin selection has revealed that although both mosquito populations have similar numbers of P450 genes that are up-regulated, for the most part the mosquito populations regulate an array of P450 genes that differ from each other. However, several P450 genes are up- and down-regulated across the two different field mosquito populations of HAmCq and MAmCq in the same way and these are distributed in families 9, 6, 4, and 325. This finding is in agreement with previous studies on the expression levels of P450 transcripts, which have often reported up-regulated expression of the P450 genes in insecticide resistant strains in *CYP* families 4, 6, and 9 [2]–[4], [9], [10], [13], [14], [16], [51]–[54] and suggested this to be a factor in the detoxification of insecticide. Unlike the previous studies, however, our study has for the first time uncovered abundant genes in CYP family 325 that are up-regulated in resistant mosquitoes in the same way as those in families 4, 6, and 9. In addition, a few of genes from clans 2 and mitochondria were up-regulated. This discovery brings new information to bear on the issue of which P450 genes and families might be involved in insecticide resistance. A previous study by our group [16] has indicated that four P450 genes, *CYP6AA7*, *CYP9J40*, *CYP9J34*, and *CYP9M10*, from mosquitoes *Cx. quinquefasciatus* are up-regulated and the overexpression levels of these four P450 genes are closely correlated to their levels of resistance, being markedly higher in HAmCq^G8^ compared to the parent strain HAmCq^G0^. The overexpression of *CYP9M10* has also been reported in a resistant *Culex* mosquito strain in Japan and has been tentatively linked with pyrethroid resistance in *Culex* mosquito [49], [50], [55]. These four P450 genes have, again, been identified as being overexpressed in resistant mosquitoes across two different field populations, strongly suggesting a common feature of these P450 genes in pyrethroid resistance in *Culex quinquefasciatus*. The significant change in the expression of these P450 genes between field parental and permethrin selected highly resistant mosquito offspring, along with the sound correlation with the levels of P450 gene expression following permethrin selection, provides a strong case further supporting the importance of these P450 genes, particularly in families 9, 6, 4, and 325, in the response to permethrin selection of resistant mosquitoes and in the development of insecticide resistance.

Our study has also revealed a down-regulation characteristic of P450 gene expression following permethrin selection in *Culex* mosquitoes. The number of down-regulated P450 genes. The clans and CYP families over which these genes were found to be distributed were similar to the up-regulated P450 genes, mainly in families 9, 6, 4 and 325. It has been pointed out that expression of many P450s is suppressed in response to various endogenous and exogenous compounds and this is also true for P450 suppression in vertebrates in response to pathophysiological signals [56]–[61]. Compared with our knowledge of P450 up-regulation involved in resistance, however, the mechanisms involved in P450 down-regulation and its relevance relating to resistance are poorly understood. It has been suggested that decreases in CYP gene expression could be an adaptive or homeostatic response [62], [63]. A number of mechanisms have been proposed for P450 down-regulation, including: 1) an adaptive homeostatic response to protect the cell from the deleterious effects of P450 derived oxidizing species, nitric oxide, or arachidonic acid metabolites [63], [64]; 2) a homeostatic or pathological response to inflammatory processes [62]; and/or 3) a need for the tissue to utilize its transcriptional machinery and energy for the synthesis of other components involved in the inflammatory response [65]. These hypotheses all offer reasonable explanations for our observation of both up- and down-regulation of multiple P450 genes in the resistant mosquitoes following permethrin selection. P450 down-regulation could, for example, be linked to the homeostatic response that insects need to protect the cell from the toxic effects of extra P450 derived oxidizing species and metabolites from the up-regulated P450s and thus balance the usage of energy, O_2_, and the other components needed for the syntheses proteins (including up-regulated P450s) that play important roles in insecticide resistance. It has been previously reported that some organophosphate insecticides require an oxidative biotransformation into more toxic structures that inhibit acetylcholinesterase, a process that is mediated by some P450 enzymes [2]. In such cases, a decrease in the expression levels of these CYP genes would be an advantage in the presence of an organophosphate insecticide by preventing its bioactivation by P450 enzymes. However, this argument may not apply to the permethrin used here for the selection of resistant mosquitoes [28], [29].

### Conclusions

The expression profiles of a total of 204 P450 genes in both larvae and adults of *Cx. quinquefasciatus* were compared between susceptible and resistant mosquito populations, two different field populations of mosquitoes, and field parental mosquitoes and their permethrin selected offspring. The results provide direct evidence that up- and down-regulation of multiple P450 genes co-occur in the genome of *Culex quinquefasciatus* following permethrin selection. These genes are mainly distributed in clans CYP3 and CYP4. These findings have important implications as they demonstrate that not only are multiple genes involved in insecticide resistance, but also multiple mechanisms are involved in P450 gene regulation. Both up- and down regulation of P450 genes may be co-responsible for the detoxification of insecticides, evolutionary insecticide selection, and the homeostatic response of mosquitoes to changing cell environments.

## Supporting Information

Table S1 Oligonucleotide primers used for amplifying the P450 qRT-PCR reactions. ^a^The transcript ID number from the vectorbase of the *Cx. quinquefasciatus* genome sequence (http://cquinquefasciatus.vectorbase.org/)^ b^The annotation of the *Culex* P450 genes from http://drnelson.utmem.edu/CytochromeP450.html [30] ^c^Specific primer pair designed according to each of the P450 gene sequences of the *Cx. quinquefasciatus* in vectorbase (http://cquinquefasciatus.vectorbase.org).
(DOC)
