# Supplementary material for: Genome Analysis of Cytochrome P450s and Their Expression Profiles in Insecticide Resistant Mosquitoes, Culex quinquefasciatus
Source: PLoS One. 2011 Dec 29;6(12):e29418. doi: 10.1371/journal.pone.0029418 (PMC3248432; doi:10.1371/journal.pone.0029418)
Supplement: Table S1 — Oligonucleotide primers used for amplifying the P450 qRT-PCR reactions. aThe transcript ID number from the vectorbase of the Cx. quinquefasciatus genome sequence (http://cquinquefasciatus.vectorbase.org/) bThe annotation of the Culex P450 genes from http://drnelson.utmem.edu/CytochromeP450.html [30] cSpecific primer pair designed according to each of the P450 gene sequences of the Cx. quinquefasciatus in vectorbase (http://cquinquefasciatus.vectorbase.org). (DOC) [file pone.0029418.s001.doc]

Table S1. Table S1. Oligonucleotide primers used for amplifying the P450 qRT-PCR reactions

| Transcript ID^a^ | Accession No. | Gene^b^ | Forward Primer (5′ to 3′)^c^ | Reverse Primer (5′ to 3′)^c^ |
| --- | --- | --- | --- | --- |
| CPIJ000067 | XM_001841684.1 | CYP49A1 | ACGGTAACGAAGAGTCCAAACGGA | TCCATGGCAATGTTGATGTGTCGC |
| CPIJ000293 | XM_001841814.1 | CYP4J18 | TTGACGCCCACGTTCCATTTCAAC | ACAAAGGTTGTAGCTCGGTGACGA |
| CPIJ000294 | XM_001841815.1 | CYP4J13 | AGCAGTTTCTGCAGGACTCGACAT | CTTCATCAATGGCGTTGTCCGCTT |
| CPIJ000298 | XM_001841819.1 | CYP6AH2 | TCCGATCATCAAGAATGTGGCGGA | ATTCCAAACGCCGAACTTGACACC |
| CPIJ000299 | XM_001841820.1 | CYP6AH3 | TCCGCAGCTTGATCGAGAATGTCA | TCAGAAACGTGACCGAGTGACCAA |
| CPIJ000655 | XM_001842366.1 | CYP329B1 | ACGTGTTCAACGTGATCTCTGGGT | TTACCGTAAACCAGCGAACTCCCA |
| *CPIJ000924 | XM_001842495.1 | CYP325X5P | AAGGGTCTCTTCACGGCCAAGTAT | CACACTTGACAAACAACGGCACGA |
| CPIJ000925 | XM_001842496.1 | CYP325X6 | TGCGCTACACCGAAATGTTCCTGA | ACCATCTTCTCGGCACACTTGACA |
| ^#^CPIJ000926 | 窗体顶端  XM_001842497.1窗体底端 | - | GCAAGCAGTACAAATTCGCCGACA | TGATCAGATCGGTCCGTTGCAAGT |
| CPIJ000927 | XM_001842498.1 | CYP325X7 | ATGCAGAACCGCCTCTTTGAGGAA | ACGCAATTGTTTCAGCATCTCGGG |
| *CPIJ000928 | XM_001842499.1 | CYP325X8P | ACAGGCGAAAGTACAGCTTCGCTA | TCAATCGATCGCTCCTGCTGAACA |
| CPIJ000929 | XM_001842500.1 | CYP325X9 | TGCTTCAACACGCGAATTCTGCAC | TCACCGACGTGTACTGGTGAATGT |
| CPIJ000989 | XM_001842607.1 | CYP307B1 | GCAGTACTTTGCCAGCCATCGTTT | TCCATGCGCTTCAGATTACGGTGA |
| CPIJ001038 | 窗体顶端  XM_001842655.1窗体底端 | CYP18A1 | TCCAAATGATGCAGGTGATTGCGG | GCAGCATAAACACGTTCAGCCACA |
| CPIJ001039 | XM_001842656.1 | CYP306A1 | GTGTTTGCCTTTGATCCTGCACCA | GGCAGGAAATTGACCGCCATTGAA |
| CPIJ001380 | XM_001843226.1 | CYP314A1 | ACTACGGGTTCAAGCTGTGGAAGT | GCTCTTCTTGCAGCGCTTCGTTTA |
| CPIJ001754 | XM_001843567.1 | CYP4J6 | ACCGTCAAGAACCTGGAGGACTTT | ATCGATCTGGTTTCGTGCTTCGGA |
| CPIJ001755 | XM_001843568.1 | CYP4J19 | CATTAAGCAACGCAGGGAGCTGTT | ATCTGATTACGTGCCTCGGATGCT |
| CPIJ001757 | XM_001843570.1 | CYP4H39 | AAAGCCACTGAGTCGTGAGGACAT | TTCTTGTCCTTGCCCAGAATCCGA |
| CPIJ001758 | XM_001843571.1 | CYP4H38 | TTACCGTTGAGGGTAAGCCGTTGA | ACAACTTCTGCTGAACGTCCTGGT |
| CPIJ001759 | XM_001843572.1 | CYP4H40 | TTTCCTGTATCCTTTCGCACGGGA | TGTAAACGTCCTCTTCCTGCTGCT |
| CPIJ001810 | 窗体顶端  XM_001843432.1窗体底端 | CYP4C38 | ACTTCAACATCCTGGACGGGTTCT | TCATGGCCGTCTCGCAGATAATGT |
| CPIJ001886 | XM_001843611.1 | CYP4H31 | TCGAATGACGTTCCTGGACCTGTT | TTCCTGCACATCTGGATGCTTTGC |
| CPIJ002535 | XM_001844152.1 | CYP6AG9 | CGTAAATGGCCTCCTGCATTTGCT | TCGGGAAAGTGATGTTCGTCCAGT |
| CPIJ002536 | XM_001844153.1 | CYP6AG10 | AACTCGCAGCCAAATTCACCACAG | TGAGTAGCTTGGCAAGTTTCGGGA |
| CPIJ002537 | XM_001844154.1 | CYP6AG11 | ACGACACTCTACTGGAATTGCCCT | CAAGTTCGATGGGTTCCGTGCATT |
| CPIJ002538 | 窗体顶端  XM_001844155.1窗体底端 | CYP6AG12 | AAGCCCTTTCAACGCTGGTTGATG | GAGCGTCGATGGCAAACACACAAT |
| CPIJ003082 | XM_001844416.1 | CYP9J42 | TGCAAGCTAGAGCTGGTCTGCTAA | TGCTTGTGCGATCATTTCCGTGTC |
| CPIJ003361 | XM_001844754.1 | CYP6BY2 | ACGGCGTTGAGAGGAATGACTTCT | ACGACGAAGTCTCGAATCCACCAA |
| CPIJ003375 | XM_001844768.1 | CYP6BY3 | ATTGGGAGTTGTGCCTTTGGCATC | TGGCCGCTTCCTCTCTAAACACTT |
| CPIJ003376 | XM_001844769.1 | CYP6BY4 | GTTGTGCAACGATTCACCACGGAT | AACAATCGCTTCTTCCCGGAATGC |
| CPIJ003377 | XM_001844770.1 | CYP6BY5 | TGGTGCTGAACTTGAGGTGAAGGA | TTCCAGCAGATATGGGCTCGTTGA |
| CPIJ003378 | XM_001844771.1 | CYP6BY6 | TTTCCTACCTTGAAGGCGGTTGGA | CAATTCCAAACGCACAGCTTCCGA |
| CPIJ003389 | XM_001844782.1 | CYP6BY7 | AGGATTACATGGTGCGGTTCACGA | ACATCCTGAACTCACTGTTCGGCT |
| CPIJ004410 | XM_001845944.1 | CYP6Z11 | TTACGGAAATATCCGGGACTGCCA | AAGTACCGTTCGTCCTTGGCGTAA |
| CPIJ004411 | XM_001845945.1 | CYP6Z12 | GCCGGCAAGAAGCTGGAACATTAT | TCCGGGTTGTTCAACGTATCCACT |
| CPIJ005332 | XM_001846722.1 | CYP9J43 | TGTGCCTTCGGTGTTAGACTGGAT | TCGATTCCAAGTTTCCCGGTGAGT |
| CPIJ005683 | XM_001847180.1 | CYP325Y10 | CTGGCAATGAACCCGAACATCCAA | AGCCGAAGCGACTCTTTGATGACT |
| CPIJ005684 | XM_001847181.1 | CYP325BB1 | ATGATCGGACCACTTCCGGTGATT | AAAGTGCGATTCAGTAGCCTCCGA |
| CPIJ005685 | XM_001847182.1 | CYP325BB2 | TCAAATGACGGAAGCGTATCGGGA | AACTCATCTTCTTCGGGTGCCTGT |
| *CPIJ005899 | XM_001847672.1 | CYP6N26P | TAATTGATCCGGACGTGTCGCAGT | AAATCATTCCGCTCAACGCGGTTC |
| CPIJ005900 | XM_001847673.1 | CYP6N23 | CGCTGTGCGTGAAACGGTTGAATA | GAAGCCGGCCAGAAAGAACACAAA |
| CPIJ005952 | XM_001847348.1 | CYP6BB4 | TACGAGCTGGCCCTTAATCCGTTT | AGACTTTCCGCAGGTGGGTACTTT |
| CPIJ005953 | XM_001847349.1 | CYP6BB3 | AGCGTTCGCTTTGACAACGACATC | TGAAGTCGTTACGCTGTGATCCGT |
| CPIJ005954 | XM_001847350.1 | CYP6CC2 | ACGTGTTCTTCTCGCCATCGATCA | AGCTTATCCCTGTACAAACCGCGA |
| CPIJ005955 | XM_001847351.1 | CYP6P14 | AAGGTGGAACCAGGTCTGACGATT | CCATCATTAGCCGCGATTGCCTTT |
| CPIJ005956 | XM_001847352.1 | CYP6BZ2 | ACCATGGCGTCAGCTAAGGATGAA | TCCGTGGTGAATCCGACTAGCAAA |
| CPIJ005957 | XM_001847353.1 | CYP6AA9 | TCACCTGTGACGTCATTGGATCGT | AGGAAGCTGTCGAAGACCCAACTT |
| CPIJ005958 | XM_001847354.1 | CYP6AA8 | AAAGGCGTTGGCGAAGAGATTCAC | TCGTCAGCAGTTCCGAACCTTCAT |
| CPIJ005959 | XM_001847355.1 | CYP6AA7 | ATGACGCTGATTCCCGAGACTGTT | TTCATGGTCAAGGTCTCACCCGAA |
| CPIJ006322 | XM_001847976.1 | CYP307A1 | AGATGGTGCGGAACTTTGACGAGA | ATCCGCTCCAGAATGAAATCCCGA |
| CPIJ006721 | XM_001849044.1 | CYP4H37v1 | ATACGACTACATCCCGTTCAGCGT | TCCGTCTTGAATCGAACATCGCGT |
| CPIJ006950 | XM_001848945.1 | CYP325BG1 | TCGGTACGCGATGATGAGCATGAA | ATATCCGTTCTGCACCCGCAGTAT |
| CPIJ006951 | XM_001848946.1 | CYP325BG2P | AGGAGCTGGTACATCAAGCGTTCA | TGTACTCGAGCTGCTTCAGCGATT |
| CPIJ006952 | XM_001848947.1 | CYP325BG3 | ACGAGCTCCAACTCGATAGGCTTT | ATGTACTCGAGCTGCTTCAGCGAT |
| CPIJ007084 | XM_001849011.1 | CYP325Z2-de2b3b | GGAGATTATTCAAGAACG | AAGTACGTCCCATCCTGTCTCAGT |
| CPIJ007085 | XM_001849012.1 | CYP325Z2 | ACGTGCGGAGTTTGGAGAAAGTGA | TCTGATCCGTGAACCCATTGCAGA |
| CPIJ007086 | XM_001849013.1 | CYP325AB1 | CTTGATGCAAATCGGGCGTGGATT | AGCTTGCGCTCGAGAATCAAATCG |
| CPIJ007089 | XM_001849016.1 | CYP325Y4 | TTGGGAATCAAGGGAGGTCTGGTT | GTGCATCCATGATCGGCACAAAGT |
| CPIJ007090 | XM_001849017.1 | CYP325Y5 | TGGCAATGCACCCAGAAATCCAAG | TTCCTTGATGACCTGCTCGGTGTA |
| CPIJ007091 | XM_001849018.1 | CYP325Y6 | AGCTACTGCTGGACGGAGTTCAAA | TCCTCAACCCAGCACTAAACGGAA |
| CPIJ007092 | XM_001849019.1 | CYP325Y7 | AAGAGGGAAGATGGCACCGAGTTT | AGACTTCGTTCATCTCGGCAACGA |
| CPIJ007093 | XM_001849020.1 | CYP325Y8 | TTGCTGCAGAAGGTGCTGATGAAC | AGAAAGCTGGTCAACATCCGGGTA |
| CPIJ007095 | XM_001849022.1 | CYP325Y9 | AAGGACGATGGAACGGGATTCACA | TCAACTCGGAAACGACCTTGTCCT |
| CPIJ007188 | XM_001849242.1 | CYP4H30 | GATGGCGTTGGACAGCATTTGTGA | AGAATGCTGAACAGCGTCGGGTAT |
| CPIJ008566 | XM_001850110.1 | CYP6Z15 | TCTCGAACGTTCCGGAGGTCAAAT | ATAGTCCTGGGTGCACTTTCGGTT |
| CPIJ008936 | XM_001850970.1 | CYP4H41 | TCCAGCAGAAGCTGTACGACGAAA | AATGCGGAGGATCTCCTTGATGGT |
| CPIJ008937 | XM_001850971.1 | CYP4H36 | CGCCGGCGTTCCACTTTAAGATTT | ATCTTGCGCGTTTATCTTCACGCC |
| *CPIJ008972 | XM_001850501.1 | CYP6F5P | CGCTTGGCGTGTACGTTCTTTGTT | ATCCTGTCGGGATACGTTGTTGCT |
| CPIJ008980 | 窗体顶端  XM_001850555.1窗体底端 | CYP301A1 | AAAGTGTACGAAGAGCTGCGACGA | TGTAAACCCGCAGAACCTCCTTGA |
| CPIJ009085 | XM_001850916.1 | CYP6AG13 | ACCTCGAGCAGGTCATCAACGAAA | CCAAAGGGAATGACCGCACAAACA |
| CPIJ009170 | XM_001850606.1 | CYP303A1 | TTCATCGTCCGGCATCTGAAGGAA | ATGAGTGCGGCAAAGTCTTGTTGG |
| ^#^CPIJ009415 | 窗体顶端  XM_001851032.1窗体底端 | - | GGCTTTGACTACGCAATGGCTGTT | TCATGTTGAAGAAGAGGTCCGGGT |
| CPIJ009468 | XM_001851364.1 | CYP4K3 | TACCAAGCAGTACAAGCGGTTCCA | AGCTTCTTCTTCCTGGTCCCACAA |
| CPIJ009469 | XM_001851365.1 | CYP4AR4 | AGAAGCGAGATGGAATCTGCCGAA | TGACCTGGTCCTTGATTTGCTCGT |
| ^#^CPIJ009471 | 窗体顶端  XM_001851367.1窗体底端 | - | AGATCGACGTGTGCAATACCCACT | TTGCGATGTTTGAACCAGTTCGGG |
| CPIJ009473 | 窗体顶端  XM_001851369.1窗体底端 | CYP4D41 | TGGCCGGAAGATGATGGAGAATCA | TTGCTCTTCTCAGCGGAGGTTTCT |
| CPIJ009474 | 窗体顶端  XM_001851370.1窗体底端 | CYP4D40 | ACCGTTTGATGTGTATCCTCGGGT | ACGTTTGACATCGCTTTAACCGCC |
| CPIJ009475 | 窗体顶端  XM_001851371.1窗体底端 | CYP4D43 | AGATCCTGGAGCAGTTTGTGGAGA | AGTCGGAGTGAATCTGTGCGTTGA |
| *CPIJ009476 | XM_001851372.1 | CYP4D44 | GGATGGTGGATCCGGACATAGTTT | ATTTGTTCGTCAGGTTGCGGTTCG |
| CPIJ009477 | XM_001851373.1 | CYP4D19 | ACCTGACGAACAAATCCGACGAGT | TCCGATCAAACACCTCCACGAACT |
| CPIJ009478 | XM_001851374.1 | CYP4D42v1 | TCAACTATCTGGTTCGGGATGCGA | ACTTCCGGCTGAGGTTCGTTATGA |
| CPIJ009569 | XM_001851144.1 | CYP326BK1 | TCAAGTGCTTGAGATGGACGGTGA | GCCAGCTGCCACAAACGTCATAAT |
| CPIJ009570 | XM_001851145.1 | CYP325BL1 | TCGATGAGGAACGTGCTCGAAAGT | TGGTGAGCAGCTGATCGATGAAGA |
| CPIJ009587 | XM_001851162.1 | CYP325K3v2 | GGCCTGCAACATGCTGGAATACAT | AATGTTGAAGATGCGCTTGCCGAG |
| CPIJ010075 | XM_001870633.1 | CYP4H35 | TTAACGCGCAGGATAATCCCGACT | TAAAGCTGTGCAGGACTGCCAATG |
| CPIJ010125 | XM_001851431.1 | CYP12F14 | TCAGCGAATGTCCGTTGAGGAAGA | TGACGTGGATCATCGTAGCACCAA |
| CPIJ010175 | XM_001870674.1 | CYP9J48 | ATGATTGCGTTCAAGCTGTTCCCG | TGGTGCTTTAGCGTACCTTTCCGA |
| CPIJ010203 | XM_001870875.1 | CYP9AM1 | AGATGTTTCAGCTAGCGCTGGGAT | ACTTCCGCCCAAATAAGCAACACG |
| CPIJ010225 | 窗体顶端  XM_001855523.1窗体底端 | CPY12F7 | TGGACAAGAATCCGAGCCAAGACA | TCTCCGGGTTCTTTGCTAAGCAGT |
| ^#^CPIJ010226 | 窗体顶端  XM_001855529.1窗体底端 | - | CGCGTTGGTCAACATTCCGAAGAA | ACGACGACCACGATGGCAAAGATA |
| CPIJ010227 | 窗体顶端  XM_001855535.1窗体底端 | CYP12F13 | TGGACAAGAATCCGAGCCAAGACA | TCTCCGGGTTCTTTGCTAAGCAGT |
| CPIJ010228 | XM_001855539.1 | CYP12F12 | AGACTACGGCGATCTGTTGGTGAT | CCGGCCGCACATTCTTTCGATAAT |
| CPIJ010229 | 窗体顶端  XM_001855544.1窗体底端 | CYP12F11 | AAGCGGCTCATGACCCTGTTAGAT | ACGCTCTGGTTGTCGTTGTTTGTG |
| CPIJ010230 | 窗体顶端  XM_001855550.1窗体底端 | CYP12F10 | ATCCGGTAATGATGCAGCCGAAGA | AGTGATGAAAGTCACCCGGCATCT |
| CPIJ010231 | 窗体顶端  XM_001855553.1窗体底端 | CYP12F9 | AGCTGGATGAGGTTTCCAGGGATT | AAAGCCAGCACTCCGATCATCTCA |
| CPIJ010272 | XM_001870820.1 | CYP325BK2 | TTCTGGAGCGCGAAGGTAGTCAAA | TGCTCGACGAACCTTGATGGTCTT |
| CPIJ010480 | XM_001854859.1 | CYP4J20 | ACGAAGAACATCGACAAGAGCCGA | GCGTCTTCGATGCAACCACTTTGA |
| CPIJ010536 | XM_001855158.1 | CYP9J44 | TGAAGACTCGGCAAGAGCAGAACA | ACTTGGGATTCTTCGACAGTGGCA |
| CPIJ010537 | XM_001855163.1 | CYP9J45 | TCAGCGGTACGGAAACGATGTGAT | AGTCCATGTTGGTCTTCTGTCCCA |
| CPIJ010538 | XM_001855166.1 | CYP9J46 | ATCCAACGTGGGAAAGTCCACTCA | TGCCGAGTTCACTGAAAGCTCGTA |
| CPIJ010539 | XM_001855170.1 | CYP9J36 | TCCACATGTTGATGGAGGTGCAGA | ATCAGCTCGTTCTCCGTCCAAACT |
| CPIJ010540 | XM_001855175.1 | CYP9J35 | TTGCCCAATGCTTCCTGTTCTTCC | GTTTCCGCCAAGTGCTTGTTCTGT |
| CPIJ010541 | XM_001855180.1 | CYP9J37 | TCTTGCTGGGAAGCCACTCAACTA | TCGTGGGAATCCATATGGTGCGAT |
| CPIJ010542 | XM_001855184.1 | CYP9J38 | AGATGCTGGATTTGCTACCGTGGA | AGGAAGAACAGGAAGCACTGGGAA |
| CPIJ010543 | XM_001855188.1 | CYP9J40 | AAAGGTACCCTGAAGCACCAGGAA | TCCAGCCAGGAAGAAGATCAAGCA |
| CPIJ010544 | XM_001855191.1 | CYP9J33 | TCCGGCCTTTACTGGTTCCAAGAT | AAAGGCACACGTCGCAATCACATC |
| CPIJ010545 | XM_001855203.1 | CYP9J41 | GATGTGATCGCAACCTGTGCGTTT | ATCCAGTCCGGAAACAGTCGGAAT |
| CPIJ010546 | XM_001855208.1 | CYP9J34 | ATCCGATGTCGGTAAAGTGCAGGT | TGTACCTCTGGGTTGATGGCAAGT |
| CPIJ010547 | XM_001855211.1 | CYP9J47 | AGGTCCAGCTTTCTCGACCAATGA | ACGGACCAACTCGTAGACTGCAAA |
| CPIJ010548 | XM_001855214.1 | CYP9J39 | AGGTACTTGTGTGTGGTTTCCGGT | TGTTTGTTCTCATCGCTGAACCGC |
| CPIJ010810 | XM_001856162.1 | CYP325BC2 | ACCGAGCGAACTTTCATCGGTACT | ACTCTTCTGGTTGATCGTCGGCAT |
| CPIJ010826 | XM_001861286.1 | CYP302A1 | GAAGCTTCGCAAGTCGCAAGAGTT | TCGCAGGTATTCCTCCAACAGTGA |
| CPIJ010858 | XM_001861680.1 | CYP6F1 | CTGAGATTGGGCAAGCTGCAAACA | ACACCTTAATACCTCCAACCGGCA |
| CPIJ011127 | XM_001861335.1 | CYP4H34 | ACCCTAAACTGTTCTGGCTCACCA | TGGATCTGTCGGTTCGGGTTCTTT |
| CPIJ011129 | XM_001861337.1 | CYP6N25 | AGCCAGATTCACGACGGATGTGAT | CTGCGCCACAACAAGCCTAAATGA |
| CPIJ011636 | XM_001861790.1 | CYP325V5v1 | TCTGTGAATTCGCTAGCCGATGGT | ATTCTTTGCAACAGCTCCGACAGC |
| CPIJ011835 | XM_001862136.1 | CYP325BM1 | AGTCCCGTTCAGCGATCAGGAAAT | TGAACTCGTCGTAAACCTTCCGCT |
| CPIJ011836 | XM_001862137.1 | CYP325BN1 | ATGTCCTTCTACGCGCTTCATCGT | TGCAGTTCCGAGAACCACCACTAA |
| CPIJ011837 | XM_001862138.1 | CYP325V2 | TTATTCCGTTCAGTGGAGGCACGA | TTCAGCAGTGCTTCAAACCGGAAG |
| CPIJ011838 | XM_001862139.1 | CYP325V3 | GCGCAAAGCGATTAATCCCACCTT | CATCTGCAAATGCTCCCAGGGTTT |
| CPIJ011839 | XM_001862140.1 | CYP325V4 | TGTTCCTCAAAGAGTGCTTGCGAC | TGTCCTTGGTTGTAAATACTG |
| CPIJ011840 | XM_001862141.1 | CYP325E4P | TTTCTTCTTCGGCAACGGGTTGGA | AATCGGTCCGAAGCGCAACTTGAA |
| CPIJ011841 | XM_001862142.1 | CYP325E3 | CAACGTGGGTCTTCGCATGTTCAA | ATTCCAGCTGCTTCTCGTGGATGA |
| CPIJ011843 | XM_001862144.1 | CYP325BH1 | GGTTTGTGGTACAACGCTGGGAAT | GCGGAAATTTGGTGTGTGGCGATA |
| CPIJ012470 | XM_001862711.1 | CYP9AL1 | TGAACGTCCTTAGGGATGGCGAAA | TTGCTAGTCGCGGAAACGAACTGA |
| CPIJ012640 | XM_001863898.1 | CYP6CP1 | CATGCGTTACGTGGATTGGTGCAT | ATTCGCGAGTCCTAGCAATGGGAT |
| CPIJ012685 | XM_001862837.1 | CYP315A1 | AACGATGCTGTGCGTCTTGGAAAC | ACTCAGGTAGCGGATGTTTGGGAT |
| CPIJ014218 | XM_001864262.1 | CYP9M10 | TGCAGACCAAGTGCTTCCTGTACT | AACCCACTCAACGTATCCAGCGAA |
| *CPIJ014219 | XM_001864263.1 | CYP9M10-de1b | TGTTTGGGACAATGTGGCCATTCG | ATAGCCCGGCTTCCTGAACGTAAA |
| CPIJ014220 | 窗体顶端  XM_001864264.1窗体底端 | CYP9M12 | TCACCGGAAGTAAGATGCGGAACA | TCCATGCCAAACGAGATTGATGCC |
| CPIJ014221 | XM_001864265.1 | CYP9M13P | TCTTCGGAGGAATCGAAAGCACGA | AATCTCCGCGTGAAGTTTGGCTTG |
| CPIJ014336 | XM_001864768.1 | CYP9M14(partial) | GTGATTCCGCCACAGGTGTTCATA | TTGGGCTACGTTCTCAGTCCAGTT |
| CPIJ014579 | XM_001864938.1 | CYP4AR3 | GATTGTGGAAACGGCCATGGGAAT | TGTCAAACATCCTCCGGAACACGA |
| CPIJ014730 | XM_001864953.1 | CYP325AA2 | GCGCAAACATCATTAACGAGCGGA | TAGGCATGGTTCGCAATTTCCTCG |
| CPIJ014939 | XM_001864989.1 | CYP305A7 | ACTTCTTCCTTCGGTTGAGGGCTT | TGATCTGGATCAGCTCGTGCAACT |
| CPIJ014940 | XM_001864990.1 | CYP305A8 | TGTTCCTCGCTGGTGGACTTAACA | TTGAGAACGATCGGACCAACGGAT |
| CPIJ014941 | XM_001864991.1 | CYP305A9 | TTGCCGTGCAGGAATTGGATGAAG | AGCTTTCGAGGTCGGTCTTCACTT |
| CPIJ014942 | XM_001864992.1 | CYP305A10 | AACGGTACTTCCACTTAGCTGCCA | TGGTCGGAACACTTCGGGATCATT |
| CPIJ014943 | XM_001864993.1 | CYP305A11 | ATGGACACTTACCACAGGTCAGCA | ACTGCCGGTTAAAGTCGACCAGTA |
| CPIJ014944 | 窗体顶端  XM_001864994.1窗体底端 | CYP15B1 | GCTGAATGTGATTTGGACGCTGCT | TTAAAGCCGGATTTGTTCGGGCAG |
| CPIJ015223 | XM_001865772.1 | CYP6F4 | CGGAAGATGGGCACCAAAGTGTTT | AGTCCTCGATGTCTTGCGCGATAA |
| CPIJ015318 | XM_001865366.1 | CYP325V5v2 | ATGACGTTCAAGAGGCTGTTTCGC | TGTACGCTTCCGTTCTAAGCTCCA |
| CPIJ015428 | XM_001865445.1 | CYP6Z10 | TGAAACACTTGCGAAGTCCAACGG | TCTGCGTTCCCTTCTTGATGACCA |
| CPIJ015681 | XM_001866290.1 | CYP4H37v2 | TGAGTGAGCTGATACTGTTGCG | TGTTTGCGTCGCTTCTGAATGACC |
| CPIJ015953 | XM_001866307.1 | CYP325BF1v2 | CTAACGGCGTGCTGGATTTCCAAT | GCAGTGTTTCGAGTTCAGCACTGT |
| CPIJ015954 | XM_001866308.1 | CYP325N3v1 | TTGAACTTGTCGTTTGGGAGTGCC | TCAAAGTTCAGCCCAAATGCCGTC |
| CPIJ015957 | XM_001866311.1 | CYP325G4 | CGAGGTGCACGCTGGATTTGATTT | AGTCTTGTGGTAAACCCAGTCCGT |
| CPIJ015958 | XM_001866312.1 | CYP325BC1 | ACATTCCGACGGTGAACAGGAAGA | TGCACGTTTATGTCCACTCCCAGA |
| CPIJ015959 | XM_001866313.1 | CYP325BJ1 | ACACGGAACAGGTCGTCAAGGAAA | GCGCTGCAGTTTGAAGATCGGAAT |
| CPIJ015960 | XM_001866314.1 | CYP325BD1 | AGTGCATTCGGAGGTCCTTCATGT | AGACTTGTCACCAGCTTATCGGCA |
| CPIJ015961 | XM_001866315.1 | CYP325BE1 | TTCCAGGAAATCCGGGACACACTT | GCATCGTTTCCTTCAGCACCATGT |
| CPIJ015963 | XM_001866317.1 | CYP325L2 | AGTACTGCAACGCAACCCGATACT | ATTCTTTCTCGATCGCATCCCGGT |
| CPIJ016284 | XM_001866492.1 | CYP4J4 | ACACGGCGAGAATACGTTATGCCT | TTGAGCTGTTCAGCCAGAATCGGT |
| *CPIJ016355 | XM_001866596.1 | CYP6AK1-de1 | GGAACTTTCTGCAGCCACTGTTGA | CAGCGCAATCCCAAACAGCTTACT |
| CPIJ016356 | XM_001866597.1 | CYP6AK1 | ATTTACGCCATCCACCGAGATCCA | TTTACCTGGAGCGTCCCAAAGTGA |
| CPIJ016846 | XM_001867236.1 | CYP6M13 | TTCAAAGTGGTGCGTGAAACGGTG | TCGAACCCAGCCAAGTAGAACACA |
| CPIJ016847 | XM_001867237.1 | CYP6CQ1 | GCGCTGAAACAGAATCGCGATCAA | TGTCCTTCCTTGGATTCGTTGGGA |
| CPIJ016848 | XM_001867238.1 | CYP6M14 | GAAATGCCAAGCCCATGGGAAGAA | GCAAAGACGCACTTCAGCAGTTCA |
| CPIJ016849 | XM_001867239.1 | CYP6M12 | TGAGGACGAAGATTTCGCCGACTT | AAACGCGCACATCCCAATCACATC |
| CPIJ016850 | XM_001867240.1 | CYP6Y4 | ACGAGTTGGGAGGAAGCACTTTGA | ACATCCAAGAAGAACTCCGCGACA |
| CPIJ016851 | XM_001867241.1 | CYP6Y5 | AAGAACACCGGAAGGTTGGAGGAA | GCGGTTGAAGAAGTGTCAAAGCCA |
| CPIJ016852 | XM_001867242.1 | CYP6N19 | ATGTCCGGGACGAAGTGGAAGAAT | ACGTCCCAATGACATCCGTGGTAA |
| CPIJ016853 | XM_001867243.1 | CYP6N21P | TATTCGACGATTACGGCGGTTGGT | TTGTTACGCAAGCTGTTGCACTCC |
| CPIJ016854 | XM_001867244.1 | CYP6N22 | ATTGGGAACTGTGCCTTTGGGTTG | TCACATCTCGCTCCATGATGGTCA |
| CPIJ016855 | XM_001867245.1 | CYP6N20v1 | CTGGCGATGAACCAAGACCTTCAA | AGGATTGATGCTGGCGGGTAGATT |
| CPIJ016856 | XM_001867246.1 | CYP6N18 | AGTGCGTAAGGGATGCTGTGAAGA | TCATGCCCTTGTGGAAGATGAGGT |
| CPIJ016857 | XM_001867247.1 | CYP6S4 | GGGCAGGTTAAAGGCCATGTTTCA | CAATCCCAAACGCACACGATCCAA |
| CPIJ017014 | XM_001868044.1 | CYP6AG14 | GCTGACGCTTTCGACGATGGATTT | ACGATTGTGGGTACTCCACGGTTT |
| CPIJ017021 | XM_001867284.1 | CYP325K3v1 | AAGGTCAAGTTTGCCGAGTCGTTG | AAATCTCGTCCGAACTCTTCCCGA |
| CPIJ017198 | XM_001867778.1 | CYP325BF1-de1b | ATGGTAGCAGGTCTTCGTGGTT | GGGTTGCGATGCAAATGGTGGAAA |
| CPIJ017199 | XM_001867779.1 | CYP325BF1v1 | AATTTAACCCGGATCGCTTCCTGC | GCAGCACGTGCACCATAATGATCT |
| CPIJ017200 | XM_001867780.1 | CYP325N3v2 | GCAAGCTTGTCCAGCAAAGGATCA | TCGCCGGTATGGAGTTCTTGTCAT |
| CPIJ017242 | XM_001867596.1 | CYP304C1 | GGTGTTCTTTGCGCTGTTCTCCAA | TTCTTGCCAACTTCGAACAGCACG |
| CPIJ017243 | XM_001867597.1 | CYP304B4 | TGTTCCCAAACGGTACCCACTACA | ATTCTCCTTGGGCTCGGTCTTCTT |
| CPIJ017244 | XM_001867598.1 | CYP304B5 | TAGCTAAAGATGTGTCCGTGCCGT | CGAATGGCCGCTGCTTCAAACTAA |
| CPIJ017245 | XM_001867599.1 | CYP304B6 | ACTGGAAGTGGAAACAGACTGCGA | CAAACAGCACATGCGGACACTTGA |
| CPIJ017246 | XM_001867600.1 | CYP304B7 | AACTGGTTATGATCCTGGCGGACT | GGCCACAAACGTTGTCGATTTCCT |
| CPIJ017351 | XM_001867494.1 | CYP4C50v1 | ATCAAATCGGTGGCATCGTCCAGA | TTTCTGCTTGCGGATCTCCTCCTT |
| CPIJ017462 | 窗体顶端  XM_001867845.1窗体底端 | CYP6E1 | TTTGGTATTGTCGGACCCTCCGTT | ACTCACATCGTTTGGTAGGACGCA |
| CPIJ017609 | XM_001867905.1 | CYP6CD3 | AGTGATGGGAAGGAAGGTGTTCCA | TTTCGTTCCTTGAGCAGTCCCGAT |
| CPIJ018494 | XM_001869103.1 | CYP6CD2 | TGGTCAAGGACTTTGGCAGCTTTG | ATCTTGCTCACGGTGAAGATCGGT |
| ^#^CPIJ018668 | 窗体顶端  XM_001869004.1窗体底端 | - | ACCAGGACATTCAGGACAGGGTTT | GCGGATACATTCGCAGCGTTTCAA |
| CPIJ018716 | 窗体顶端  XM_001868893.1窗体底端 | CYP4C38 | CTGCTCGAGGAGAAGAGTGTCATT | CCGCGTAATTAGCTCGTGGTTGAT |
| CPIJ018854 | 窗体顶端  XM_001869293.1窗体底端 | CYP4C50v2 | ATCAAATCGGTGGCATCGTCCAGA | TTGTTGGACAGTTTCTGCTTGCGG |
| CPIJ018943 | 窗体顶端  XM_001869165.1窗体底端 | CYP4C52v1 | CACGCCGGCATTCCACTTTAAGAT | GGATTCCCATGGCGGTTTCACAAA |
| CPIJ018944 | XM_001869166.1 | CYP4C51v1 | TCGTGAACCGTCCATGATCGAACT | TTTCCACCTGGACATCCTCGGTTA |
| CPIJ019395 | XM_001869675.1 | CYP4C52v2 | TGGAGGAACAGCTTGGAAATCGGA | TTAGCTGGTGGGCTCGAACGTATT |
| *CPIJ019586 | XM_001869902.1 | CYP6Z13P | GAAGAGTGCGCAGCGAATGTGAAT | AGCTGATCTCACCACCTGATTGCT |
| CPIJ019587 | XM_001869903.1 | CYP6Z14 | AGTGATTTCGGGCTCTCGTTGGAA | TGAACCTTCTGCATGAGATCCGGT |
| CPIJ019673 | XM_001869873.1 | CYP6AG15 | CAGCGACAATGAGTTTGCCGAGTT | TTAGTCATACGCGACGCAAGCTCT |
| CPIJ019700 | XM_001870137.1 | CYP6M15 | GCGTGTTTGCGAAGGACTTTCAGT | CGACAACTTGTGCCGCAGATTCTT |
| CPIJ019701 | XM_001870138.1 | CYP6CQ2 | ACCGATTCCGGTGTTGATTCGAGA | ACGGTTCGCGTTTGGACTTTGATG |
| CPIJ019702 | XM_001870139.1 | CYP6M16 | GAAATGCCAAGCCCATGGGAAGAA | GCAAAGACGCACTTCAGCAGTTCA |
| CPIJ019703 | XM_001870140.1 | CYP6Y6 | AAGAACACCGGAAGGTTGGAGGAA | TTCCCTGTTCATGGCGAGTTCGTA |
| CPIJ019704 | XM_001870141.1 | CYP6N24 | TGTCATTGGAACGTGTGCGTTTGG | AAATCCATCGTCGAAAGCGTCAGC |
| CPIJ019705 | XM_001870142.1 | CYP6N27P | GCACAGCTGCTACGAACAACTCAA | AGCGAGCGGTCGTGAAAGTACAAA |
| CPIJ019751 | XM_001869920.1 | CYP6AG16 | TGGAGCCATCGTTTGGATCTCTGT | GCCCGTTTGATTGAGTTTGCCTCA |
| CPIJ019765 | 窗体顶端  XM_001869935.1窗体底端 | CYP9M14 | TGACTCAGTGCAAGATCCGGACAA | AATATGAACACCTGCGGCGGAATC |
| CPIJ020018 | XM_001870574.1 | CYP6Z16 | TGTCCAAGTTTCGGTTCGAGGCTA | AGGTGATGGCATCCGTTGAGGTAT |
| CPIJ020019 | XM_001870603.1 | CYP6Z17 | TGGAATCTGTGAAGGCAGACCACA | TTCCGTTGGTCAAGATCTGCGTGT |
| CPIJ020082 | XM_001870617.1 | CYP6F6 | ACCTGGAGAACTGCATCGACGAAA | AGTACTGGGATGATGACGGTGGTT |
| CPIJ020199 | XM_001870352.1 | CYP6N20v2 | GCTGTTGGATGTGTTCAAGCCGTT | AGCACCTTCACGCTCTTTCGGTAA |
| CPIJ020229 | XM_001870408.1 | CYP4D42v2 | GAGCTTCAAATTTGGCCCGCCATT | ACTTCCGGCTGAGGTTCGTTATGA |

^a^The transcript ID number from the vectorbase of the *Cx. quinquefasciatus* genome sequence [<http://cquinquefasciatus.vectorbase.org/>]

^b^The annotation of the *Culex* P450 genes from <http://drnelson.utmem.edu/CytochromeP450.html> [36]

^c^Specific primer pair designed according to each of the P450 gene sequences of the *Cx. quinquefasciatus* in vectorbase (http://cquinquefasciatus.vectorbase.org)

*Pseudogene <http://drnelson.utmem.edu/CytochromeP450.html>

^#^No annotation in Dr. Nelson’s P450 homepage <http://drnelson.utmem.edu/CytochromeP450.html>
